# Haploidentical transplantation in patients with multiple myeloma making use of natural killer cell alloreactive donors

**DOI:** 10.1007/s00277-020-04303-z

**Published:** 2020-10-28

**Authors:** Catharina Van Elssen, Gwendolyn van Gorkom, Christine Voorter, Peter von dem Borne, Ellen Meijer, Lotte Wieten, Gerard Bos

**Affiliations:** 1 Internal Medicine, Division of Hematology, Maastricht University Medical Center, P. Debyelaan 25, 6229 HX, Maastricht, Netherlands; 2 Department of Transplantation Immunology, Maastricht University Medical Center, Maastricht, Netherlands; 3 Department of Hematology, Leiden University Medical Center, Leiden, Netherlands; 4 Department of Hematology, Amsterdam University Medical Center, Location VUMC, Cancer Center, Amsterdam, Netherlands

**Keywords:** Stem cell transplantation, Multiple myeloma, NK cells

## Abstract

Disease relapse is an important problem after allogeneic stem cell transplantations in multiple myeloma (MM). To test the hypothesis that natural killer (NK) cell alloreactivity in the setting of a haploidentical stem cell transplantation (haploSCT) can reduce the risk of myeloma relapse, we performed a small prospective phase 2 study in which we transplanted poor-risk MM patients using a killer cell immunoglobulin-like receptor (KIR)-ligand mismatched haploidentical donor. Patients received bone marrow grafts after reduced-intensity conditioning, with post-transplantation cyclophosphamide (PTCY) graft-versus-host-disease (GVHD) prophylaxis. The primary endpoint was 1.5-year progression-free survival (PFS); stopping rules were installed in case interim results made a benefit of 50% PFS at 1.5 years unlikely. After inclusion of 12 patients, of which 9 were evaluable for the primary endpoint, all patients relapsed within a median time of 90 days. All except 1 patient showed engraftment, with a median time to neutrophil recovery of 18 (12–30) days. The study was prematurely terminated based on the predefined stopping rules after the inclusion of 12 patients. With this small study, we show that in chemo-resistant myeloma patients, NK cell KIR-mismatch is not superior to conventional alloSCT. This strategy, however, can serve as a platform for new treatment concepts.

Clinical Trial Registry: NCT02519114

## Introduction

In recent years, the use of haploidentical donor cells for the treatment of hematological malignancies has been increasing and has proven effective and safe, especially with the use of PTCY [1, 2]. There is limited documentation about haploSCT in MM, but small retrospective studies show this is feasible with relatively low non-relapse mortality (NRM). Results in terms of PFS are however similar to HLA-identical SCT [3–5]. HaploSCT offers an attractive opportunity to introduce natural killer (NK) cell alloreactivity based on a KIR-ligand mismatch. In myeloid malignancies, this NK alloreactivity, in the context of a T cell deplete haploSCT, leads to a decreased relapse rate and improved survival without causing GVHD [6–8]. Unlike in T cell–depleted haploSCT, the benefit of NK cell alloreactivity in T cell replete haploidentical SCT is less clear and the limited number of small-sized studies concerning this effect is not conclusive [9–12]. The differences concerning NK cell alloreactivity in these studies might be due to the disease heterogeneity of the included patients and differences in the conditioning regimen and stem cell source. Some studies point towards a better outcome when bone marrow stem cells were used compared to peripheral blood stem cells, which was shown to correlate with the T cell content of the graft that is higher in peripheral blood stem cell products [12]. This correlation is in line with reports on the inhibitory effect of T cells on the development of alloreactive NK cells [13].

There is much evidence to support the role of NK cells in fighting MM [14–17]. It has been proven that therapeutic interventions like lenalidomide and elotuzumab result in increased NK cell–mediated anti-myeloma responses [18, 19]. In preclinical studies performed by our research group, we show that NK cells are able to kill MM cells, and this killing is improved in the presence of NK alloreactivity [20, 21]. This was shown in vitro as well as in a humanized mouse model. Two small clinical studies also implicated a beneficial role for KIR-ligand mismatch in MM. In one study, the role of administration of alloreactive NK cells before autologous SCT was examined. High remission rates were observed, although they were short-lived [22]. In another study, the impact of KIR-ligand mismatch in a HLA-identical and mismatched SCT setting was investigated and showed that KIR-ligand mismatch in the graft-versus-host direction was protective for relapse [23].

The aim of this phase 2 study was to prospectively evaluate if KIR-ligand mismatched haploidentical bone marrow transplantation (BMT) with PTCY improves PFS in poor-risk MM patients.

## Methods

### Patients

In this prospective, single-arm, multicenter trial, we recruited poor-risk MM patients aged below 66 years with good clinical performance from hospitals in the Netherlands. Poor risk was defined as high-risk cytogenetics (del17p and/or *t*(4;14) and/or *t*(14;16)), or relapse within a year after autologous SCT, or relapse after three or more previous lines of therapy. Furthermore, patients had to be responsive to their last line of therapy, defined as at least partial response according to the International Myeloma Working Group consensus criteria [24]. Another prerequisite of enrolment was the permissiveness to NK alloreactivity and availability of a KIR-ligand mismatched haploidentical family donor. Patients were excluded if donor-specific HLA-antibodies were present.

### Donor selection

All patients were transplanted with a KIR-ligand mismatched haploidentical family donor. The opportunity for KIR-ligand mismatched haploBMT was determined by Luminex sequence-specific oligonucleotide hybridization (SSO) typing for the three possible inhibitory KIR-ligands: HLA-C group 1 (ligands for KIR2DL2/3), HLA-C group 2 (ligands for KIR2DL1), and HLA-Bw4 including HLA-A harboring Bw4 motifs as ligands for KIR3DL1 (A*23, A*24, A*32).

In case of an opportunity for KIR-ligand mismatched haploBMT, a KIR-ligand mismatched haploidentical family donor was searched in the wide family tree of the patients. In case a probable KIR-ligand mismatched donor was identified by low resolution, a second blood sample was drawn from this potential donor for confirmation in high resolution, for KIR typing, by low-resolution Luminex SSO assay. Protein expression of the mismatched KIR was confirmed by immune phenotyping of the peripheral blood NK cells for KIR expression as described below.

### Immune phenotyping for KIRs during donor selection and NK cell reconstitution

Peripheral blood mononuclear cells were isolated by gradient density centrifugation and stained with monoclonal antibodies with specificity for CD3 (SK7, BD), CD56 (B159, BD), NKG2A (Z199, Beckman Coulter), KIR2DL1 (143211, R&D), KIR2DL2/3/S2 (DX27, Miltenyi Biotech), or KIR3DL1 (DX9, Miltenyi Biotech) followed by acquisition of the samples on a BD FACS Canto II machine. Acquired data were analyzed using the Diva software by gating on living CD3- CD56+ lymphocytes followed by analysis of the percentage of positive cells for the individual KIRs.

### Conditioning and transplant procedure

Conditioning regimen consisted of cyclophosphamide 14.5 mg/kg on day - 6 and - 5, fludarabine 30 mg/m^2^ from day - 6 to - 2 and 200 cGY total body irradiation at day - 1 in all but one patient that received busulfan instead of cyclophosphamide pre-transplant. Donor bone marrow cells were infused on day 0. Bone marrow cells were used in all but one patient, since they are preferred over peripheral blood stem cells because of a lower risk of acute and chronic GVHD [25, 26].

### GVHD prophylaxis and supportive care

GVHD prophylaxis consisted of cyclophosphamide 50 mg/kg at day + 3 and + 4. Mycophenolate mofetil was used from day + 5 to + 35. Tacrolimus 0.1 mg/kg was added to this combination from day + 5 to + 180.

To prevent infections, patients received immunoglobulins 0.2 g/kg once every 4 weeks from 1 week before conditioning until the immunosuppressive drugs were stopped. Anti-microbial prophylaxis furthermore consisted of cotrimoxazole and valaciclovir, and during neutropenia, ciprofloxacin and fluconazole were given as selective digestive decontamination.

### Study endpoints and statistical analysis

The primary endpoint was PFS at 1.5 years. Since haploBMT is a demanding and costly treatment for the patients, we considered the effect that has to be realized by this procedure needed to be substantial and chose for the PFS goal of 50% in 1.5 years compared to around 25% with conventional alloSCT according to historical data.

To test this hypothesis, Simon’s two-stage design was used. We hypothesized that the 1.5-year PFS will be 50% after haploBMT, while this is a maximum 25% in the hypothetical standard treatment group (conventional alloSCT according to historical data). To demonstrate this difference with a power of 80% and a type 1 error rate, alpha (one-sided) of 0.05%, 24 patients were needed. If 1 or less of the first 9 patients experienced 1.5-year PFS, the relevant predefined positive effect was considered very unlikely and the study would be stopped.

Secondary endpoints were engraftment, bone marrow reconstitution, NK cell reconstitution and repertoire, GVHD, infections, and NRM.

To ensure safety, we build in decision rules to prematurely terminate the study if the NRM at 100 days exceeded a certain percentage that was calculated beforehand based on a modification of the standard 3 + 3 scheme.

Analyses were performed as of April 2020. Pre-transplantation patient characteristics were given as median and range for continuous variables and as frequency and proportion for categorical variables. PFS was analyzed using a Kaplan-Meier estimate. For NRM, relapse, and GVHD, a competing risk framework was used. The analysis was performed with the R software.

## Results and discussion

In total, 12 poor-risk patients were included from 3 hospitals in the Netherlands from April 2016 to May 2018 with a follow-up until April 2020 and the median time to follow-up is 30.2 months (range 11.8–44.9 months). Pre-transplantation patient characteristics are described in Table 1. They were all heavily pre-treated with both proteasome inhibition and immunomodulatory drugs; none of the patients received antibody treatment before inclusion. One patient had known high-risk cytogenetics and three patients showed progression within 12 months after autologous SCT. We excluded one patient for further analysis due to disease progression just before BMT, since this was a predefined exclusion criterion. This patient, however, was transplanted because pre-transplant M-protein levels became available during conditioning therapy. KIR/HLA incompatibility of the different donors is described in Table 2.

**Table 1:** Patient characteristics

Gender, *n* (%)	
Male	10 (91)
Female	1 (9)
Age, median in years (range)	61 (40–66)
Response to last therapy, *n* (%)	
PR	5 (45)
VGPR	5 (45)
CR	1 (9)
Previous lines of treatment, median (range)	3 (2–7)
Previous SCT, *n* (%)	
1x autologous	8 (73)
2x autologous	3 (27)
Allogeneic	1 (9)

**Table 2:** Donor and recipient HLA typing and NK mismatch

Patient NR.	Patient HLA present	Patient HLA absent	Donor HLA present	Donor HLA absent	Mismatch	iKIR mismatch
P1	Bw4, C1	C2	Bw4, C1, C2	X	C2	2DL1
P2	C2	Bw4, C1	C1, C2	Bw4	C1	2DL2/2DL3
P3	Bw4, C2	C1	Bw4, C1, C2	x	C1	2DL2/2DL3
P4	C1, C2	Bw4	Bw4, C2	C1	Bw4	3DL1
P5	Bw4, C1	C2	C1, C2	Bw4	C2	2DL1
P6	C1	Bw4, C2	Bw4, C1, C2	X	C2, Bw4	2DL1/3DL1
P7	Bw4, C1	C2	Bw4, C1, C2	X	C2	2DL1
P8	C1, C2	Bw4	Bw4, C1, C2	X	Bw4	3DL1
P9	Bw4, C2	C1	Bw4, C1, C2	X	C1	2DL2/2DL3
P10	C1	C2,Bw4	Bw4, C1, C2	X	C2, Bw4	2DL1/3DL1
P11	C1	C2, Bw4	Bw4, C1, C2	X	C2, Bw4	2DL1/3DL1
P12	C1, Bw4	C2	Bw4, C1, C2	x	C2	2DL1

*HLA* human leukocyte antigen, *iKIR* inhibitory killer cell immunoglobulin-like receptor

### Clinical endpoints

Of the 11 evaluable patients, 10 achieved primary engraftment (91%), with a median time to neutrophil and platelet engraftment of 18 (12–30 days) and 30 (20–49 days) days, respectively. Grade 2–4 acute GVHD occurred in 2 of 11 patients (none grade 3–4) and chronic GVHD occurred in 4 of 11 patients. Two of the 11 patients died of treatment-related mortality (18%) within the first year.

**Fig. 1:**
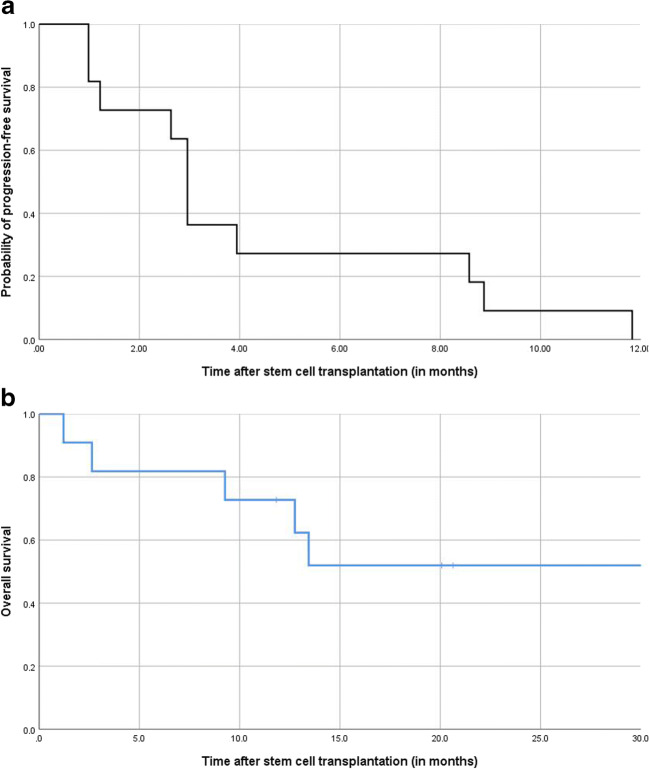
Clinical outcomes after HaploBMT. **a** Probability of progression-free survival. **b** Overall survival

Of the 9 for the primary endpoint evaluable patients, all patients relapsed within 1 year (Fig. 1a). The median time to relapse was 90 days (range 30–360 days), and 8 of 9 patients had to eventually start anti-myeloma treatment. The median time for the next treatment was 186 days (range 40–330 days); two-thirds of the patients at first only biochemically relapsed without a need for treatment. Overall survival was 73% after 1 year and 52% after 2 years (Fig. 1b).

**Fig. 2:**
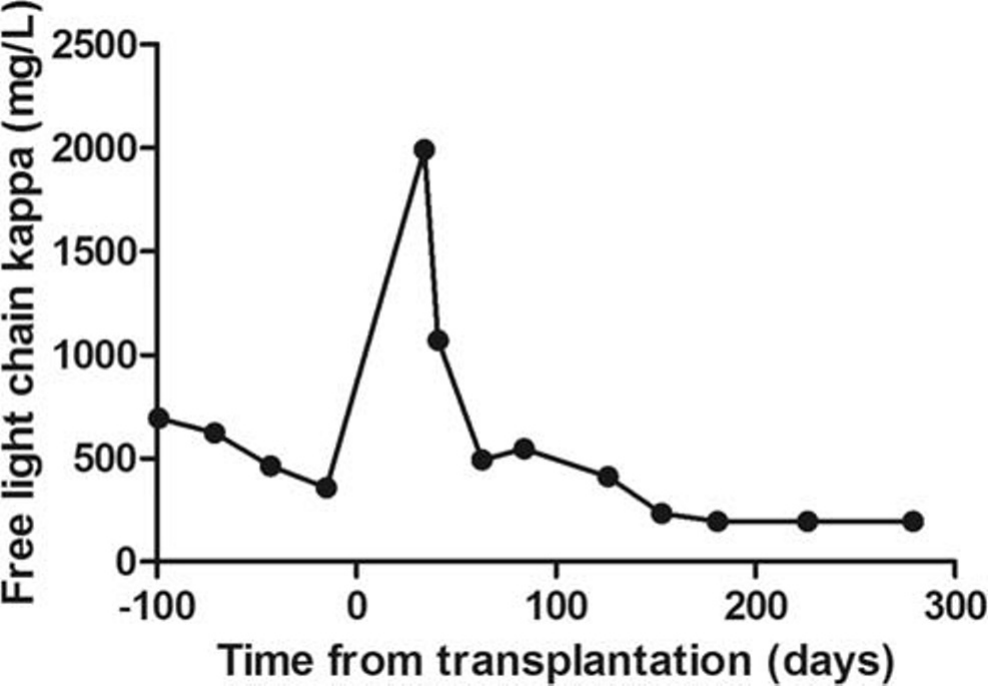
Serum-free light-chain response after HaploBMT in one individual patient. Progression 30 days after transplantation and responsive thereafter without treatment

Noteworthy is one patient, who relapsed quickly after SCT with an increase of her involved free light chain, displayed a spontaneous decrease 60 days after BMT to pre-transplant levels (Fig. 2). During this period, no additional anti-myeloma treatment was started. Only immune-suppressive treatment with mycophenolate mofetil was stopped at day + 35, as per protocol. She did not require any treatment until 400 days after the stem cell transplantation. Interesting is also a second, heavily pre-treated patient that was already progressive at day 40 after SCT, but had a complete remission on daratumumab and has continuous bone marrow–proven remission more than 2 years after stopping this treatment.

### NK cell reconstruction

**Fig. 3:**
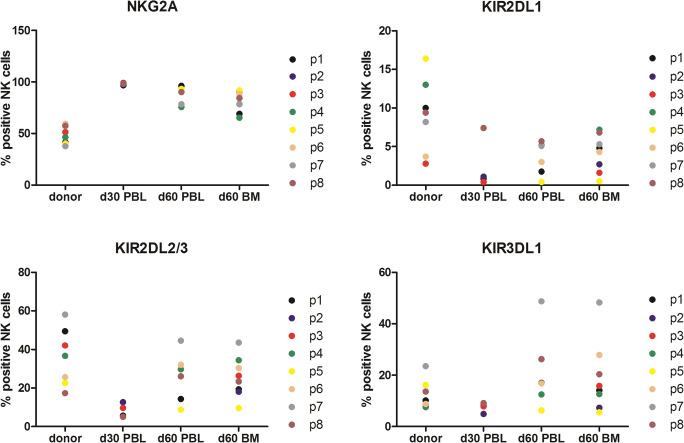
**a**–**d** Assessment of NK cell phenotype after stem cell transplantation. Mononuclear cells were isolated from peripheral blood (PBL) or bone marrow (BM). To analyze KIR expression in the donor, PBL were harvested before from the donor before transplantation. To determine NK reconstitution upon transplantation, PBL or BM cells were isolated from the patient at 30 (d30) or 60 (d60) days after transplantation. Isolated cells were stained and the percentage of CD3-CD56+ NK cells expressing KIR2DL1, KIR2DL2/3, KIR3DL1, or NKG2A was determined by flow cytometry. Patients and their donors are depicted as P1–P8 in the legend and every dot represents one data point of cells analyzed at day 30 (d30) or day 60 (d60) after transplantation

At day 30, all of the 8 analyzed patients showed NK cell recovery, though with an immature phenotype (NKG2A^+^, KIR^-^). At day 60 in both the peripheral blood and bone marrow, mature NK cells (KIR^+^) could be identified (Fig. 3a–d). We have no data on the functionality of these cells.

## Discussion

With this study, we tested the safety and the efficacy of a KIR-mismatched haploidentical BMT in high risk MM patients. We observed that the treatment is safe since there was a high engraftment rate, low NRM (18% at 1 year), and no unexpected adverse events. In the only two other retrospective studies concerning haploSCT with PTCY in MM where there was no selection on KIR-mismatch, comparable results were seen with respect to NRM (10–21% in 1.5 to 2 years); however, they had a slightly higher PFS (17–33% in 1.5 to 2 years). In our small study, unfortunately, there is a 1.5-year PFS of 0%. This 1.5-year PFS seems lower than in conventional alloSCT studies. However, the study is underpowered to draw these conclusions, since the study was prematurely terminated due to the defined stopping rules based on a 1.5-year PFS of 50%. Furthermore, this low PFS was also not unexpected since these were heavily pre-treated patients with very high-risk chemo-resistant myelomas. Though these patients showed a biochemical relapse, many of them did not require treatment for a long time after which is very unlikely for this fast progressive patient population. Even though it is difficult to draw conclusions in such a small, heterogenous group of patients, our results show that KIR-ligand mismatch in this patient category is not only harmful but also not more effective than non-matched haploSCT or conventional alloSCT in curing MM [3, 4].

We hypothesize that the late reconstitution of functional mature NK cells is responsible for the lack of response. This has already been described in other patient groups after PTCY: within days after SCT, mature graft-derived NK cells appear, but they are rapidly eliminated by PTCY. Full reconstitution of a mature and functional NK cell population took 6 to 12 months [12]. The median time point of relapse in our patients occurred shortly after transplantation (around 3 months). We detected that 30 days after haploBMSCT, most NK cells still had an immature phenotype without KIR expression and were not able to stop disease progression at this early stage after transplantation when myeloma cell load was still low. We hypothesize that the delayed NK cell reconstitution may be overruled by the infusion of mature, functional donor NK cells shortly after PTCY administration. In myeloid malignancies, this concept has already been used and seems successful in a small group of high-risk patients [27]. In this context, it may also be helpful to bridge the time to full NK cell restoration after the SCT with additional anti-MM therapy like elotuzumab or daratumumab, or to combine these two modalities. This concept is supported by the patient that showed a long-lasting complete response after temporary treatment with daratumumab. Another option would be to improve NK cell function and thereby increase the alloreactive effect by blocking antibodies, such as monalizumab, a novel checkpoint inhibitor against NKG2a [28].

In conclusion, in this study, we show that haploidentical BMT in MM patients is safe and feasible in terms of engraftment and late NK cell reconstitution. HaploBMT in MM forms a possible platform for future immunotherapeutic strategies in which the KIR-ligand mismatch might be beneficial. Though this study is limited in patient numbers, none of the patients showed a clinically relevant increase of PFS; therefore, we conclude that in the setting of haploBMT in combination with PTCY, however, a KIR mismatch probably is not clinically relevant.

## References

[1] Luznik L, O'Donnell PV, Symons HJ, Chen AR, Leffell MS, Zahurak M, Gooley TA, Piantadosi S, Kaup M, Ambinder RF, Huff CA, Matsui W, Bolaños-Meade J, Borrello I, Powell JD, Harrington E, Warnock S, Flowers M, Brodsky RA, Sandmaier BM, Storb RF, Jones RJ, Fuchs EJ (2008). HLA-haploidentical bone marrow transplantation for hematologic malignancies using nonmyeloablative conditioning and high-dose, posttransplantation cyclophosphamide. Biol Blood Marrow Transplant.

[2] McCurdy SR, Kasamon YL, Kanakry CG, Bolanos-Meade J, Tsai HL, Showel MM (2017). Comparable composite endpoints after HLA-matched and HLA-haploidentical transplantation with post-transplantation cyclophosphamide. Haematologica..

[3] Sahebi F, Garderet L, Kanate AS, Eikema DJ, Knelange NS, Alvelo OFD, Koc Y, Blaise D, Bashir Q, Moraleda JM, Dreger P, Sanchez JF, Ciurea S, Schouten H, Shah NN, Verbeek M, Rösler W, Diez-Martin JL, Schoenland S, D'Souza A, Kröger N, Hari P (2019). Outcomes of haploidentical transplantation in patients with relapsed multiple myeloma: an EBMT/CIBMTR report. Biol Blood Marrow Transplant.

[4] Castagna L, Mussetti A, Devillier R, Dominietto A, Marcatti M, Milone G, Maura F, de Philippis C, Bruno B, Furst S, Blaise D, Corradini P, Montefusco V (2017). Haploidentical allogeneic hematopoietic cell transplantation for multiple myeloma using post-transplantation cyclophosphamide graft-versus-host disease prophylaxis. Biol Blood Marrow Transplant.

[5] Chen Y, Lu J, Xu LP, Chen H, Zhang XH, Wang FR, Chen YH, Wang Y, Liu KY, Huang XJ (2018). Safety and efficacy of haploidentical stem cell transplantation for multiple myeloma. Bone Marrow Transplant.

[6] Ruggeri L, Capanni M, Urbani E, Perruccio K, Shlomchik WD, Tosti A (2002). Effectiveness of donor natural killer cell alloreactivity in mismatched hematopoietic transplants. Science (New York, NY).

[7] Pende D, Marcenaro S, Falco M, Martini S, Bernardo ME, Montagna D, Romeo E, Cognet C, Martinetti M, Maccario R, Mingari MC, Vivier E, Moretta L, Locatelli F, Moretta A (2009). Anti-leukemia activity of alloreactive NK cells in KIR ligand-mismatched haploidentical HSCT for pediatric patients: evaluation of the functional role of activating KIR and redefinition of inhibitory KIR specificity. Blood..

[8] Giebel S, Locatelli F, Lamparelli T, Velardi A, Davies S, Frumento G, Maccario R, Bonetti F, Wojnar J, Martinetti M, Frassoni F, Giorgiani G, Bacigalupo A, Holowiecki J (2003). Survival advantage with KIR ligand incompatibility in hematopoietic stem cell transplantation from unrelated donors. Blood..

[9] Wanquet A, Bramanti S, Harbi S, Furst S, Legrand F, Faucher C (2018). Killer cell immunoglobulin-like receptor-ligand mismatch in donor versus recipient direction provides better graft-versus-tumor effect in patients with hematologic malignancies undergoing allogeneic T cell-replete haploidentical transplantation followed by post-transplant cyclophosphamide. Biol Blood Marrow Transplant.

[10] Bastos-Oreiro M, Anguita J, Martinez-Laperche C, Fernandez L, Buces E, Navarro A (2016). Inhibitory killer cell immunoglobulin-like receptor (iKIR) mismatches improve survival after T-cell-repleted haploidentical transplantation. Eur J Haematol.

[11] Willem C, Makanga DR, Guillaume T, Maniangou B, Legrand N, Gagne K, Peterlin P, Garnier A, Béné MC, Cesbron A, le Bourgeois A, Chevallier P, Retière C (2019). Impact of KIR/HLA incompatibilities on NK cell reconstitution and clinical outcome after T cell-replete haploidentical hematopoietic stem cell transplantation with posttransplant cyclophosphamide. J Immunol.

[12] Russo A, Oliveira G, Berglund S, Greco R, Gambacorta V, Cieri N, Toffalori C, Zito L, Lorentino F, Piemontese S, Morelli M, Giglio F, Assanelli A, Stanghellini MTL, Bonini C, Peccatori J, Ciceri F, Luznik L, Vago L (2018). NK cell recovery after haploidentical HSCT with posttransplant cyclophosphamide: dynamics and clinical implications. Blood..

[13] Cooley S, McCullar V, Wangen R, Bergemann TL, Spellman S, Weisdorf DJ, Miller JS (2005). KIR reconstitution is altered by T cells in the graft and correlates with clinical outcomes after unrelated donor transplantation. Blood..

[14] Osterborg A, Nilsson B, Bjorkholm M, Holm G, Mellstedt H (1990). Natural killer cell activity in monoclonal gammopathies: relation to disease activity. Eur J Haematol.

[15] Frohn C, Hoppner M, Schlenke P, Kirchner H, Koritke P, Luhm J (2002). Anti-myeloma activity of natural killer lymphocytes. Br J Haematol.

[16] El-Sherbiny YM, Meade JL, Holmes TD, McGonagle D, Mackie SL, Morgan AW (2007). The requirement for DNAM-1, NKG2D, and NKp46 in the natural killer cell-mediated killing of myeloma cells. Cancer Res.

[17] Carbone E, Neri P, Mesuraca M, Fulciniti MT, Otsuki T, Pende D, Groh V, Spies T, Pollio G, Cosman D, Catalano L, Tassone P, Rotoli B, Venuta S (2005). HLA class I, NKG2D, and natural cytotoxicity receptors regulate multiple myeloma cell recognition by natural killer cells. Blood..

[18] Campbell KS, Cohen AD, Pazina T (2018). Mechanisms of NK cell activation and clinical activity of the therapeutic SLAMF7 antibody, elotuzumab in multiple myeloma. Front Immunol.

[19] Giuliani M, Janji B, Berchem G (2017). Activation of NK cells and disruption of PD-L1/PD-1 axis: two different ways for lenalidomide to block myeloma progression. Oncotarget..

[20] Sarkar S, Germeraad WT, Rouschop KM, Steeghs EM, van Gelder M, Bos GM (2013). Hypoxia induced impairment of NK cell cytotoxicity against multiple myeloma can be overcome by IL-2 activation of the NK cells. PLoS One.

[21] Sarkar S, Noort W, van Elssen C, Groen R, van Bloois L, van Gelder M, Schouten H, Tilanus M, Germeraad W, Wieten L, Martens A, Bos G (2015). Alloreactive Natural Killer cells have anti-tumor capacity against disseminated human multiple myeloma in Rag2-/-γC-/-mice when combined with low dose cyclophosphamide and total body irradiation. HSAO J Clin Immunol Immunother.

[22] Shi J, Tricot G, Szmania S, Rosen N, Garg TK, Malaviarachchi PA, Moreno A, DuPont B, Hsu KC, Baxter-Lowe LA, Cottler-Fox M, Shaughnessy Jr JD, Barlogie B, van Rhee F (2008). Infusion of haplo-identical killer immunoglobulin-like receptor ligand mismatched NK cells for relapsed myeloma in the setting of autologous stem cell transplantation. Br J Haematol.

[23] Kroger N, Shaw B, Iacobelli S, Zabelina T, Peggs K, Shimoni A, Nagler A, Binder T, Eiermann T, Madrigal A, Schwerdtfeger R, Kiehl M, Sayer HG, Beyer J, Bornhauser M, Ayuk F, Zander AR, Marks DI, the Clinical Trial Committee of the British Society of Blood and Marrow Transplantation and the German Cooperative Transplant Group (2005). Comparison between antithymocyte globulin and alemtuzumab and the possible impact of KIR-ligand mismatch after dose-reduced conditioning and unrelated stem cell transplantation in patients with multiple myeloma. Br J Haematol.

[24] Kumar S, Paiva B, Anderson KC, Durie B, Landgren O, Moreau P, Munshi N, Lonial S, Bladé J, Mateos MV, Dimopoulos M, Kastritis E, Boccadoro M, Orlowski R, Goldschmidt H, Spencer A, Hou J, Chng WJ, Usmani SZ, Zamagni E, Shimizu K, Jagannath S, Johnsen HE, Terpos E, Reiman A, Kyle RA, Sonneveld P, Richardson PG, McCarthy P, Ludwig H, Chen W, Cavo M, Harousseau JL, Lentzsch S, Hillengass J, Palumbo A, Orfao A, Rajkumar SV, Miguel JS, Avet-Loiseau H (2016). International Myeloma Working Group consensus criteria for response and minimal residual disease assessment in multiple myeloma. Lancet Oncol.

[25] Nagler A, Dholaria B, Labopin M, Savani BN, Angelucci E, Koc Y et al (2020) Bone marrow versus mobilized peripheral blood stem cell graft in T-cell-replete haploidentical transplantation in acute lymphoblastic leukemia. Leukemia 34:2766–2775 10.1038/s41375-020-0850-9 32393841

[26] Ruggeri A, Labopin M, Bacigalupo A, Gulbas Z, Koc Y, Blaise D (2018). Bone marrow versus mobilized peripheral blood stem cells in haploidentical transplants using posttransplantation cyclophosphamide. Cancer..

[27] Ciurea SO, Schafer JR, Bassett R, Denman CJ, Cao K, Willis D, Rondon G, Chen J, Soebbing D, Kaur I, Gulbis A, Ahmed S, Rezvani K, Shpall EJ, Lee DA, Champlin RE (2017). Phase 1 clinical trial using mbIL21 ex vivo-expanded donor-derived NK cells after haploidentical transplantation. Blood..

[28] van Hall T, Andre P, Horowitz A, Ruan DF, Borst L, Zerbib R (2019). Monalizumab: inhibiting the novel immune checkpoint NKG2A. J Immunother Cancer.

